# Mentosternal Contracture Treated With an Occipito-Scapular Flap in a 5-year-old Boy: A Case Report

**Published:** 2008-02-15

**Authors:** Armin Kraus, Hans-Eberhard Schaller, Hans-Oliver Rennekampff

**Affiliations:** Department for Hand, Plastic, Reconstructive and Burn Surgery, BG Trauma Center, Eberhard Karls Universität Tübingen, Germany

## Abstract

*Objective:* Mentosternal contracture, a complication of deep thermal injury to the neck, results in significant functional and esthetic problems. A new method of reconstructing this deformity is described.

*Methods:* We present a case of a 5-year-old patient with severe mentosternal contracture, treated with a large occipito-scapular island flap (24 cm × 10 cm) augmented by anastomosing the circumflex scapular artery to the facial artery.

*Results:* A 46% functional improvement in the range of motion was achieved, and an esthetically pleasing appearance was obtained.

*Conclusions:* This case report demonstrates the occipitocervical dorsal flap to be an excellent option for mentosternal contracture release. Microvascular flap perfusion augmentation should be considered even in cases of limited vascular dimensions as is often seen in the pediatric patient population.

## BACKGROUND

The cervico-mental area is perhaps the most important esthetic and functional area in burn patients. The natural neck posture places the head in the most optimal alignment for daily interactions. Despite well-planned multidisciplinary support for burn survivors, severe contracture of the neck frequently complicates the early postinjury course of the patients who suffer from thermal injuries to the head and neck. Neck reconstruction for cervical scar contracture after burn injuries often takes priority over other areas. Airway access and protection, critical for subsequent surgical intervention, make this zone particularly important and should be addressed early. Neck contracture when suffered in childhood and left untreated inhibits normal mandibular growth.^1^ Adjacent areas such as the face and shoulders are further drawn into the contractual deformity with resultant functional deformation to the mouth and periorbita.

There are essentially 3 separate categories of neck contracture for which different surgical concepts are advocated.^2^ Isolated vertically oriented scar bands can usually be released with Z-plasties. Scar contracture involving burned skin with intervening normal skin can be treated with incisional release and skin grafting or local flaps. Local unburned tissue can be expanded and recruited to provide improved color and skin texture. Severe neck contracture involving most of the neck skin generally requires excision and release of the platsyma, and the entire esthetic unit of the neck should be addressed to improve the functional and esthetic result. Local island flaps as well as free flaps may also provide well-vascularized tissue to resurface large area defects.^3^–^5^

The concept of microvascular augmented island flaps has been successfully employed to resurface extensive areas after burn scar excision in adults.^3^,^5^ We describe the case of an occipito-scapular flap with microsurgical anastomosis to the facial artery in a 5-year-old boy with severe mentosternal contracture after burn injury.

## CASE REPORT

**Figure 1:**
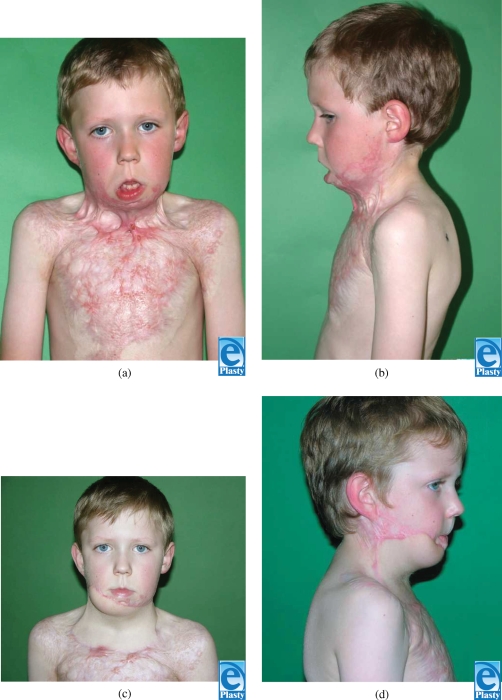
Preoperative view (a). A severe neck contracture with extrinsic contractures of the face was observed, range of motion was poor. Lateral view (b) showing severe neck contracture grade E3 as described by Tsai.^3^ Postoperative appearance (c, d). Range of motion has improved with complete release of extrinsic contractures with a good posture at rest.

A 5-year-old boy suffered from severe flame burn injury to neck and chest. Despite early excision and thick skin grafts as well as postoperative splinting, compression, and occupational therapy, he developed a grade E3 (severe) mentosternal contracture, as described by Tsai.^3^ Objective measurements noted flexion contractures of 0°/20°/30°, a maximum chest-chin distance of 3 cm, rotation to right and left at 20°, and extrinsic depression of the right commissure (Figs 1a and 1b).

**Figure 2:**
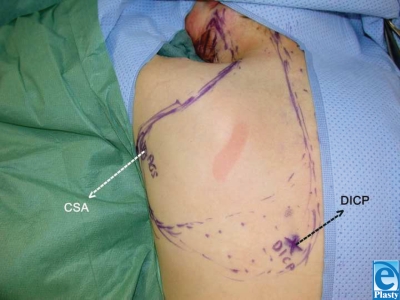
Preoperative view. Outline of the flap is marked. Circumflex scapular artery (CSA) and one dominant dorsal intercostal perforator (DICP) are marked.

**Figure 3:**
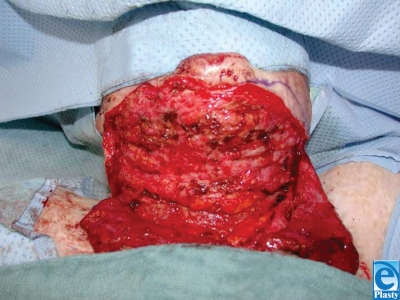
Intraoperative view of mental region. The contracture is released by an incision at the cranial border of the scarred area.

**Figure 4:**
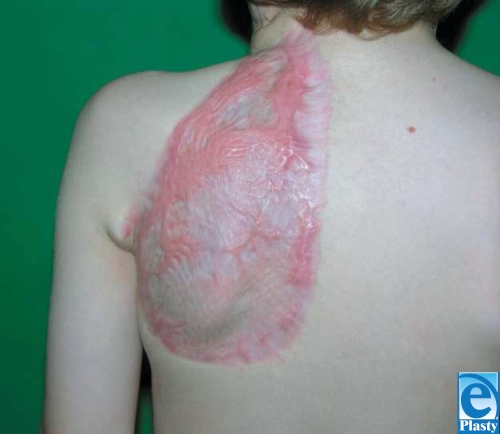
Late postoperative view of the donor site 6 months after surgery.

A left-sided occipito-scapular dorsal flap was designed to reconstruct the anterior neck for contracture release and resurfacing. The defect area and requisite flap dimension was 24 cm × 10 cm. Vascular augmentation with a microvascular anastomosis of the circumflex scapular artery and dorsal intercostal perforator (DICP) was incorporated into the preoperative design to enhance viability. Preoperative color Doppler ultrasonography was performed to map out 7th and 9th DICPs (Fig. 2). Surgical dissection of the circumflex scapular artery and DICP revealed that the DICP was too small for reliable anastomosis. The scar was excised and the neck released with the patient in the supine position (Fig. 3). The flap was harvested in the prone position, and the circumflex scapular artery anastomosed to the right facial artery in the supine position. The donor site was covered with split-thickness skin grafts (Fig. 4). On postoperative day 5, a small area of a 3 cm × 1 cm with inadequate perfusion was excised and primarily closed. At 6-month follow-up (Figs 1c and 1d), head reclination testing demonstrated a maximum chin-sternum distance of 8 cm (7 cm at rest), and head rotation was measured at 55° to the left and 50° to the right.

## DISCUSSION

The goals of treatment for severe neck contracture are to release the contracture, restore the contour of the mentosternal angle, and prevent recontracture. In our case, typical criteria of advanced contracture grading is evident by involvement of both upper cervical and lower cervical areas in the deformity.^3^ Activities of daily living, such as school attendance, participation in sport, and social interactions, are impaired and may pose additional psychological stress on the developing child. This 5-year-old boy was in preschool. Risk and benefit concerns were discussed with the family who elected to have the functional and esthetic deformities addressed before entry into school.

We prefer to include full-thickness platsyma transection with scar excision to best define the cervico-mental angle and to minimize the risk of recontracture. In some cases, a complete release is not possible because of scarring of underlying tissue such as muscle. In addition, size and length of the obtained flap may not fully match the scarred area. A pleasing cervical profile relies on a well-defined cervico-mental angle, defined in adults as between 105° and 126°.^6^ In this case of a young child, we obtained a 100° angle nearly achieving the ideal.

Scar release was performed by incision of the scarred area at the cranial border of the scar. Care must be taken not to injure the mandibular branch of the facial nerve (cranial nerve VII) when dissecting the submental area.

Many methods have been advocated to reconstruct severe neck contracture, including split-thickness and full-thickness skin grafts, dermal substitutes combined with skin grafts, local or pedicle flaps with or without tissue expanders, pedicle or free myocutaneous flaps, and free septocutaneous flaps. Skin grafts alone or in combination with a dermal substitute like Integra have been described with varying success^7^ Tissue expanders can increase skin territory; however, maximum skin gain after tissue expansion is generally about 25%.^8^,^9^ Local or pedicle skin flaps with similar color, thickness, and texture are limited to the territory of uninvolved skin. In our case, both shoulders had significant scarring precluding the use of an expanded supraclavicular artery island flap.

Free as well as pedicle myocutaneous flaps have been successfully used for neck release with subsequent debulking procedures often advocated. Free fascio-cutaneous or perforator flaps provide a better alternative.^3^ In a young child, limited donor site size and regional morbidity minimize suitability of flaps like the radial forearm and lateral thigh perforator flaps. Tsai et al^3^ have described a series of burn neck reconstructions with free tissue transfer, the youngest patient being 8 years old. Reoperation was necessary in 5% of their cases, a complication that may be minimized with the use of island flaps.

Hyakusoku^5^,^10^ pioneered the field of extended vascular-augmented and thinned cutaneous island flap. Ogawa and Hyakusoku^5^ further defined the expected survival area of various dorsal scapula flaps with a narrow skin pedicle over the superficial cervical artery and vein and incorporating additional vascular contributions from the circumflex scapular artery and/or lateral DICP. As suggested by Ogawa et al,^11^ we could identify a dominant DICP by color Doppler ultrasonography preoperatively. Upon intraoperative dissection, a DICP vessel diameter of less than 1 mm was found and considered too small for reliable microvascular anastomosis to the transverse cervical artery or superior thyroid artery.

Initial donor site morbidity with a skin-grafted area at the back has to be mentioned. This can be addressed at a later stage by skin expansion of adjacent uninjured skin should the patient so desire. In the meantime, the donor site is hidden by most clothing.

Functional and esthetic reconstruction of the contracted neck is essential for quality of life. The thinned occipito-scapular flap with microvascular augmentation is an excellent option for single-stage reconstruction of severe neck contracture. With appropriate patient selection and preoperative planning, this procedure can be safely employed in the pediatric patient.
